# Divide and Conquer: A Flexible Deep Learning Strategy for Exploring Metabolic Heterogeneity from Mass Spectrometry Imaging Data

**DOI:** 10.1021/acs.analchem.2c04045

**Published:** 2023-01-12

**Authors:** Lei Guo, Jiyang Dong, Xiangnan Xu, Zhichao Wu, Yinbin Zhang, Yongwei Wang, Pengfei Li, Zhi Tang, Chao Zhao, Zongwei Cai

**Affiliations:** † Department of Electronic Science, National Institute for Data Science in Health and Medicine, Xiamen University, Xiamen, Fujian 361005, China; ‡ Bionic Sensing and Intelligence Center, Institute of Biomedical and Health Engineering, Shenzhen Institute of Advanced Technology, Chinese Academy of Sciences, Shenzhen, Guangdong 518055, China; § State Key Laboratory of Environmental and Biological Analysis, Department of Chemistry, Hong Kong Baptist University, Hong Kong SAR 999077, China; ∥ School of Mathematics and Statistics, The University of Sydney, Sydney, NSW 2006, Australia; ⊥ School of Artificial Intelligence, Beijing Normal University, Beijing 100875, China; # Department of Oncology, The Second Affiliated Hospital of Medical College, Xi’an Jiaotong University, Xi’an, Shaanxi 710004, China; ∇ Bruker Scientific Technology Co., Ltd., Beijing 100086, China; ○ School of Public Health, Dongguan Key Laboratory of Environmental Medicine, Institute of Environmental Health, Guangdong Medical University, Dongguan, Guangdong 523808, China

## Abstract

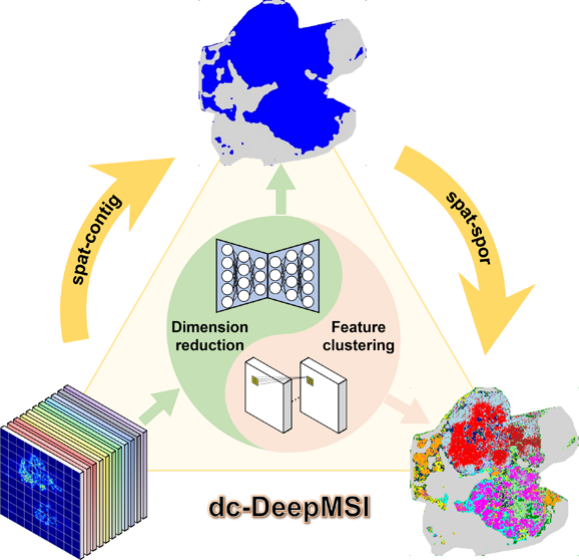

Research on metabolic heterogeneity provides an important basis for the study of the molecular mechanism of a disease and personalized treatment. The screening of metabolism-related sub-regions that affect disease development is essential for the more focused exploration on disease progress aberrant phenotypes, even carcinogenesis and metastasis. The mass spectrometry imaging (MSI) technique has distinct advantages to reveal the heterogeneity of an organism based on *in situ* molecular profiles. The challenge of heterogeneous analysis has been to perform an objective identification among biological tissues with different characteristics. By introducing the divide-and-conquer strategy to architecture design and application, we establish here a flexible unsupervised deep learning model, called divide-and-conquer (dc)-DeepMSI, for metabolic heterogeneity analysis from MSI data without prior knowledge of histology. dc-DeepMSI can be used to identify either spatially contiguous regions of interest (ROIs) or spatially sporadic ROIs by designing two specific modes, spat-contig and spat-spor. Comparison results on fetus mouse data demonstrate that the dc-DeepMSI outperforms state-of-the-art MSI segmentation methods. We demonstrate that the novel learning strategy successfully obtained sub-regions that are statistically linked to the invasion status and molecular phenotypes of breast cancer as well as organizing principles during developmental phase.

## Introduction

Mass spectrometry imaging (MSI) could provide a plethora of metabolic information directly from biological specimens on disease research, including spatial distribution, abundance, and composition of thousands of biomolecules.^1,2^ Identification from MSI data of the regions of interest (ROIs), which are statistically linked sub-regions or biologically functional regions, is usually used to differentiate cell types from heterogeneous tissue and in turn to contribute to our understanding of the cellular specificity of tissue,^3,4^ and it allows better targeting of the lesions and distant metastases that are associated with disease diagnosis and prognosis.^5,6^ In particular, ROI analysis has become a critical foundation, allowing for subsequent detection of known biomarkers and discovery of unknown biomarkers with a major focus in tumor research.^7^ Nevertheless, the fundamental question of how to improve accuracy and specificity of ROI analysis is not crystal clear.

Segmentation is the common method for ROI analysis in MSI data, which is accomplished by clustering data points (MSI image pixels) with similar characteristics into a cluster (*i.e.*, ROI). An effective segmentation result means that each cluster could link to a sub-region with a specific molecular phenotype, and the difference between clusters on MSI data can be used to interpret the biological heterogeneity on the tissue.^8^

MSI segmentation by far is a challenging task because of the complexities of MSI data featured in high-dimensional spectra, low signal-to-noise ratios, and lack of benchmark datasets.^9^ The existing methods for MSI segmentation can be roughly divided into supervised and unsupervised depending on whether prior knowledge of ROI labels is used. In supervised methods, data from histopathology, pathology, or other imaging modals like MRI are often evident in the ROI labels of MSI pixels and then guided to train the segmentation model of MSI data.^10,11^ However, MSI data is of much more molecular information, which can shape some “hidden” sub-regions that might not be differentiated from histological or other imaging techniques; therefore, segmentation results will be biased if supervised by histological data or other imaging modals. Nevertheless, some MSI studies lack prior knowledge of full ROI labels because of the extremely precious tissue specimens; in such cases, the supervised methods cannot be used. On the contrary, unsupervised segmentation is an exploratory approach in which no prior information is needed for pixel clustering, so the unsupervised segmentation is more practical and gains more extensive attention than the supervised one in MSI segmentation.

Dozens of unsupervised methods have been proposed for MSI segmentation in the past decades. For example, Abdelmoula *et al*. use t-distributed stochastic neighbor embedding (t-SNE) to reduce the dimensionality of MSI data and then use *k*-means to segment MSI data into a certain number of clusters that are expected to be in coincidence with the prognostic tumor subpopulations,^12^ the widely used vendor software^13^ SCiLS Lab uses *k*-means to conduct MSI segmentation on some selected ions rather than on extracted features, the Cardinal package provides a new unsupervised clustering algorithm, namely spatial shrunken centroids, to produce a smooth MSI segmentation,^14^ and so on. To our best knowledge, most of the existing unsupervised methods apply statistical model-based clustering algorithms like *k*-means to identify ROIs from MSI data. Since model-based clustering algorithms usually rely on a certain mathematical hypothesis of ROI,^15^ for example, *k*-means assume that data points from the same cluster are high-dimensional spherical distribution around the ROI center,^16^ model-based clustering algorithm would fail to identify the ROIs that are unsatisfied with its underlying hypothesis. However, as we know, MSI datasets of tissue with complex diseases like tumor are highly heterogeneous, that is, data points from different sub-regions might distribute in-homogeneously across the MSI dataset. Thus, different sub-regions are of specific discriminate validities under a certain model-based clustering algorithm, making the segmentation results be poorly determined. It is urgent to develop a flexible clustering algorithm which is adaptive to the high heterogeneity of MSI data.

Deep learning is flourishing in recent years and achieved great success in various fields especially for biomedical image analysis. Deep learning features a data-driven strategy and the ability of learning automatically the multiscale structure from the data,^17^ which allows us to develop a flexible and adaptive clustering algorithm for MSI segmentation. Although deep learning-based methods have been proposed for some contexts of MSI data analysis like classification,^18,19^ the deep learning-based unsupervised segmentation for MSI segmentation is rarely reported, which can be attributed to two main challenges including the intrinsic high dimensionality of MSI data and the sensitivity of unsupervised deep learning methods in parameter initialization.

Here, we propose a flexible deep learning-based method called divide-and-conquer (dc)-DeepMSI for segmentation of MSI data by introducing the dc strategy into model designation, training, and application. The task of MSI segmentation is divided into two separated sub-tasks, namely, dimensionality reduction and feature clustering, and then two independent modules are designed and trained to conquer the two sub-tasks and to meet with the above-mentioned challenges accordingly. In addition, dc-DeepMSI provides two specific modes to adapt to different types of ROI segmentation, the general mode (spat-contig) and the specific mode (spat-spor). We illustrate the feasibility of dc-DeepMSI in two typical applications: experimental results show that dc-DeepMSI outperforms state-of-the-art MSI segmentation methods, which successfully identifies 12 different organs from a whole-body mouse fetus MSI image and effectively explores the metabolic heterogeneity from a human breast tumor MSI image. Biomarker screenings are performed on the ROIs identified by dc-DeeepMSI from the tumor tissue, which further demonstrate that dc-DeepMSI can be used to detect the ROIs connected with clinical diagnosis and thereby helping illuminate the metabolism-associated diseases.

## Experimental Procedures

### Sample Collection

The whole-body mouse fetus at embryonic day 18 and human breast cancer samples are collected for MALDI-MSI analysis. The procedures of animal experiments are approved by the Institutional Animal Care and Use Committee at Shenzhen Institutes of Advanced Technology, Chinese Academy of Sciences. The volunteer is recruited with consent and handled in accordance with approved procedures from the Institutional Review Board of the Second Affiliated Hospital of Medical College, Xi’an Jiaotong University and Shenzhen Institutes of Advanced Technology, Chinese Academy of Sciences (no. YSB-2021-Y0213). The details of experiments are showed in Material S1.

### Data Acquisition

The tissue samples are sectioned at a 14 μm thickness by using a CryoStar Nx79 cryostat (Thermo Fisher Scientific, Germany). MSI datasets are collected by using a RapifleX MALDI Tissuetyper (Bruker Daltonics, Germany). Detailed information of two MSI datasets are provided in Table S1. An H&E image is acquired by using a Nanozoomer 2.0RS digital pathology scanner (Hamamatsu, Japan). More details of MALDI-MSI and histological analysis can be found in the previous work of Zhao *et al*.^20,21^

### Data Preprocessing

SCiLS Lab vendor software is used to read and export MSI data to imzML files. The MALDIquant package is used to carry out data preprocessing including spectral alignment, peak detection, and peak binning.^22^ Then, a data matrix *X*_*M* × *N* × *H*_ is obtained for further analysis, in which *M* and *N* are pixel numbers of horizontal and vertical coordinates of the MSI image, respectively, and *H* is the hyperspectral dimensionality, or say the ion (*m/z*) number.

### Architecture of dc-DeepMSI

**Figure 1:**
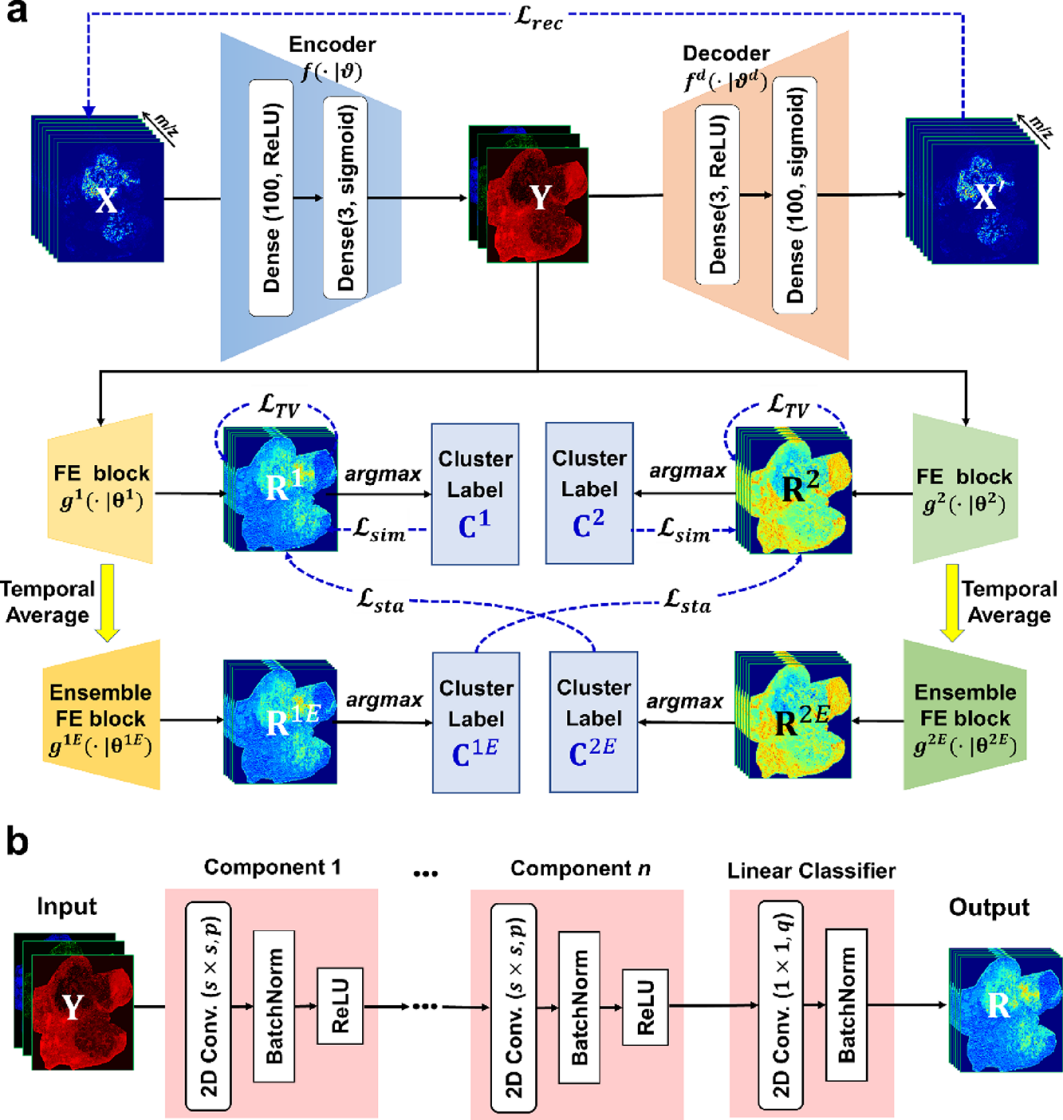
Schematic overview of dc-DeepMSI. (a) Architecture of dc-DeepMSI. The upper half part is the DR module, which is implemented by an autoencoder. The lower half part is the FC module, which consists of two CNNs and their temporally ensemble copies. (b) Architecture of FE block. An FE block consists of *n* CNN components and a linear classifier, in which the CNN component consists of a 2D convolutional layer with *s* × *s* kernel size and *p* filters, a batch normalization layer, and a ReLU activation function; the linear classifier consists of a 2D convolutional layer with 1 × 1 kernel size and *q* filters and a batch normalization layer.

By introducing the divide-and-conquer strategy into the deep neural network, a deep learning model named dc-DeepMSI is proposed here for unsupervised segmentation of high-dimensional MSI data, in which the task is divided into two independent sub-tasks, dimensionality reduction (DR) and feature clustering (FC), as shown in Figure 1a. The DR module (the upper panel of Figure 1a) is implemented by an autoencoder.^23^ The FC module (the lower panel of Figure 1a) is designed as two CNNs and their temporally ensemble copies. Two CNNs are structurally identical with independent parameter initialization; the output of one ensemble CNN feeds into the other CNN network, and vice versa, with the intent to reduce the randomness and to achieve stable feature clustering. The CNNs here play the roles of feature extraction (FE) and *argmax* classification (Figure 1b). The architecture of dc-DeepMSI including a loss function, activation function, and implementation are detailed in Materials S3 and S4.

In addition, by setting two hyper-parameters, *i.e.*, the convolutional kernel size *s* and the weight of total variation (TV) loss ω_3_ (Material S4, eq S16), dc-DeepMSI can switch its working modes between the general mode of spat-contig (*s* > 1, ω_3_ > 0) and the specific mode of spat-spor (*s* = 1, ω_3_ = 0) to meet with different ROI scenarios in a variety of specimens. spat-contig mode is designed for spatially contiguous ROI identification, while spat-spor mode is designed for spatially sporadic ROI identification. Nevertheless, the two modes are not antagonistic. Spatially sporadic ROIs can also be successfully identified by the general mode of spat-contig with a small ω_3_.

### Lipid Ion Screening Method

Four commonly used metrics, including Hedges’ *g* effect size (ES),^24^ fold change (FC), area under the curve (AUC), and least absolute shrinkage and selection operator (LASSO) regression,^25^ are used to screen lipid markers for each sub-region in the breast tumor sample. Details of the four metrics and the screening methods can be found in Material S2.

## Results and Discussion

### dc-DeepMSI Identifies Sub-organs of Mouse Fetus *via* the General spat-contig Mode

**Figure 2:**
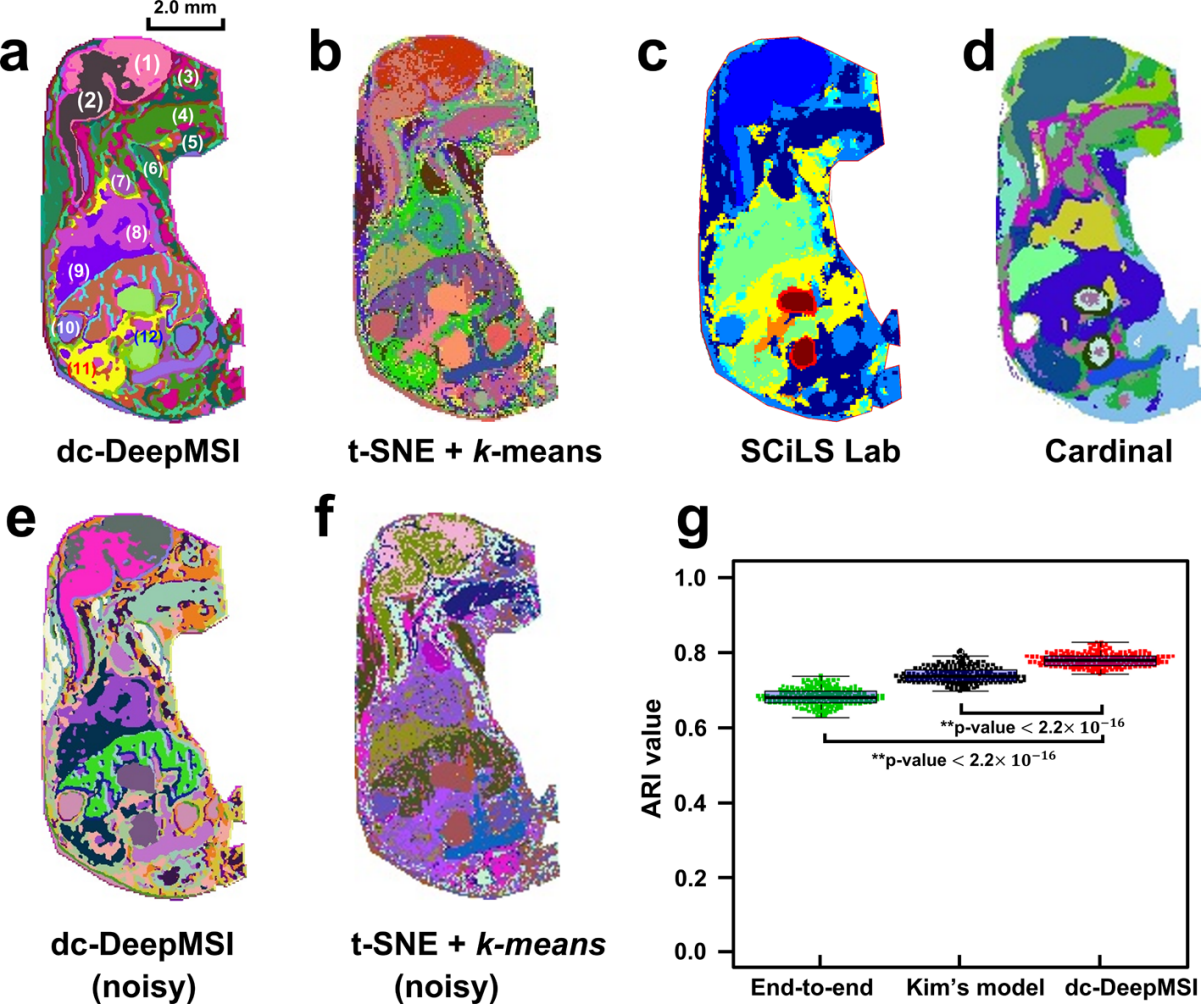
Identification of sub-organs of mouse fetus. (a–d) Color-encoded segmentation maps obtained from dc-DeepMSI, t-SNE + *k*-means, SCiLS Lab, and Cardinal on original MSI data. Compared with the other three methods, dc-DeepMSI shows a smoothing clustering result as well as a better resolution of sub-organs. (e, f) Segmentation maps obtained from dc-DeepMSI and t-SNE + *k*-means on a noisy MSI data. (g) Comparison of ARI values of an end-to-end model, Kim *et al.*’s model, and dc-DeepMSI model, where Wilcoxon rank-sum tests are carried out on the ARI values of these methods. The organs and sub-organs are as follows: (1) dorsal pallium (isocortex) and hippocampal formation (Hpf) regions, (2) midbrain and brainstem, (3) orbital cavity, (4) genioglossus muscle, (5) submaxillary gland, (6) sternebra, (7) thymus, (8) heart, (9) liver, (10) adrenal gland, (11) kidney, and (12) intestine.

The ROIs, in which data points contiguously distribute across the dataset, are a common scenario in MSI data of various biological tissues. Among them, MSI images of whole-body mouse fetus are a typical example with such spatially contiguous ROIs. Most noteworthy, molecular features and organ identification of mouse fetus are considered to be the complex and critical preprocess with applications in areas such as embryological genetics, pathology, and pharmacology.^26^ Due to limitations in terms of technology, we have not been able to profile the multi-organ structures of the mouse fetus from MSI images. To address this issue, we construct a dc-DeepMSI model on the MSI data of the mouse fetus (embryonic day18) to identify organs and their sub-organs, in which the general mode spat-contig is adopted in view of the spatial continuity of MSI pixels from the same ROI. A total of 12 organs are identified by dc-DeepMSI including the brain (1 and 2 in Figure 2a), orbital cavity (3), genioglossus muscle (4), submaxillary gland (5), sternebra (6), thymus (7), heart (8), liver (9), adrenal gland (10), kidney (11), and intestine (12) (Figure 2a). More importantly, functional sub-organ structures are recognized from the whole brain organ, such as the dorsal pallium (isocortex) and hippocampal formation (Hpf) region, midbrain, brainstem, and cerebellum (Figure 2a).

To illustrate the performance of dc-DeepMSI on segmentation of MSI data, three commonly used methods are carried out on the MSI dataset of the mouse fetus for comparison, including t-SNE + *k*-means implemented by the Python library Scikit-learn,^12^ a pipeline provided by commercial SCiLS Lab software,^20^ and a pipeline implemented by Cardinal package.^27^ The segmentation map of the t-SNE + *k*-means method shows abundant isolated clusters and obscure boundaries on mouse fetus, especially on fetal brain, which might due to the lack of a spatial denoising procedure in the method (Figure 2b). SCiLS Lab software tends to segment some big-size organs, such as brain and thoracic cavity, while it fails to identify the sub-organs (Figure 2c). Segmentation results of the Cardinal package is a little bit better than SCiLS Lab software but still miss some sub-organs, such as in the brain of the mouse fetus, where the dorsal pallium (isocortex) and Hpf regions are clearly distinct. (Figure 2d). The failure of sub-organ identification might be due to the adoption of feature selection instead of feature extraction in a dimension reduction procedure in SCiLS Lab and Cardinal package, which may result in severe information loss. In addition, the comparison experiment on computational time also illustrates the better performance of dc-DeepMSI (Table S2). These results demonstrate that dc-DeepMSI outperforms the other three methods in more and accuracate organ/sub-organ analysis.

Robustness and stability are two pivotal indicators for deep learning-based methods.^28^ Here, simulated Poisson noise is generated and added on the MSI data for robustness evaluation, and then dc-DeepMSI and the other three conventional methods are carried out on the noisy data. dc-DeepMSI shows its robustness against the noise by identifying accurately most of the organs and sub-organs from the noisy fetus data, for example, the brain and its sub-organs (Figure 2e). The t-SNE + *k*-means method meanwhile delivers too many isolated clusters on the segmentation map (Figure 2f). Sensitivity to parameters initialization is another nuisance in most of deep learning-based methods.^29^ Here, we design a comparative experiment to illustrate the model stability of dc-DeepMSI and two other deep learning models, including an end-to-end architecture model without an explicit dimension reduction module (Figure S2a**)** and a deep model proposed in Kim *et al.*’s work (Figure S2b).^30^ Twenty times of training with different parameters initialization are carried out, and the adjusted rand index (ARI) values are calculated (Figure 2g). The end-to-end model has a small *ARI mean* = 0.68 and a large ARI standard deviation (*std* = 0.020), which implies the high sensitivity of parameter initialization. Kim *et al.*’s model improves its stability by dimension reduction module architecture (*ARI mean* = 0.74, *std* = 0.021), while dc-DeepMSI is of the best model stability (*ARI mean* = 0.78, *std* = 0.017) because of the divide-and-conquer strategy and double-CNN structures. More detailed evaluation results can be found in Materials S5 and S6 and Table S3.

Above all, dc-DeepMSI is more suitable for segmentation on high-heterogeneity and high-dimensional data analysis with outstanding robustness and stability.

### dc-DeepMSI Explores Metabolic Heterogeneity of Human Breast Tumor

Being different from the organ identification depending on spatially contiguous ROIs, some MSI datasets suggest that cellular distribution is characterized by the sporadic arrangement as well as diversity on morphology, such as human tumors, biofilms, and single-cell imaging.^6,12,31^ To specify the dc-DeepMSI application on spatially sporadic ROI detection, taking the human breast sample as an example, the specific mode of dc-DeepMSI is carried out on intratumor regions to explore tumor metabolic heterogeneity. Thus, another divide-and-conquer-based strategy is leveraged by dc-DeepMSI on application, in which the MSI dataset of a complex tumor sample is divided into cancerous and para-carcinoma regions using the general mode of dc-DeepMSI, and then exploring of tumor metabolic heterogeneity is conquered using the specific mode of dc-DeepMSI. The image from hematoxylin and eosin (H&E) staining (Figure S3) is used as a simple tool to assist the interpretation of histological information for MSI dataset partially.

#### Cancerous and Para-carcinoma Discriminating *via* the General spat-contig Mode

**Figure 3:**
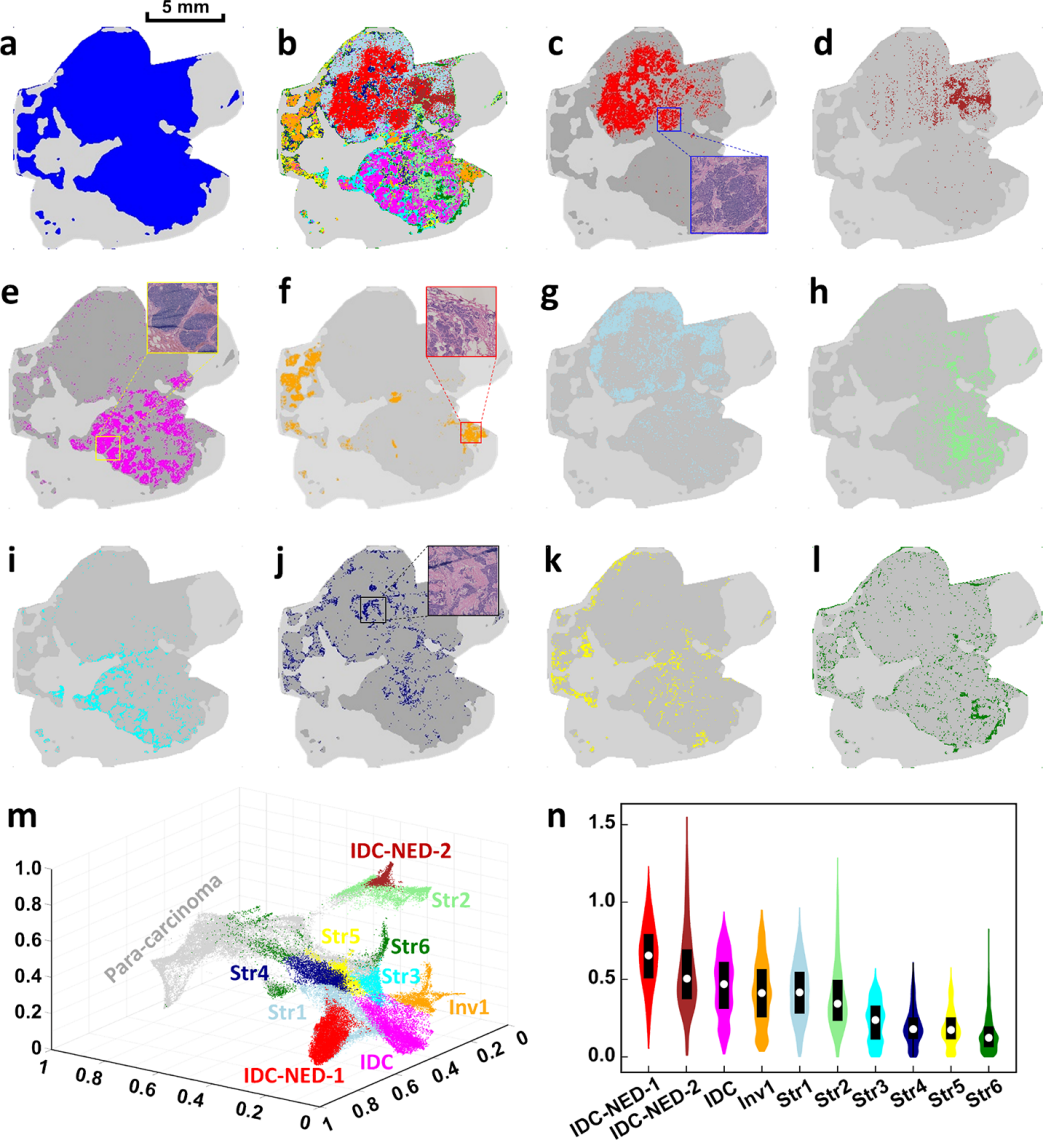
Results of dc-DeepMSI on human breast cancer data. (a) Cancerous and para-carcinoma regions. (b) Intact tumor sample. (c, d) Invasive ductal carcinoma with NED-related sub-regions, called IDC-NED-1 and IDC-NED-2, respectively. (e) Invasive ductal carcinoma-related sub-regions, called IDC. (f) Typical invasive region. (g–l) Stromal regions 1–6, called str 1–6. (m) Scatter plot of data points in embedding space corresponding to (b). (n) Violin plot of Euclidean distances between data points of each sub-region in the cancerous region and data points of the para-carcinoma region in the embedding space.

Cancerous cells from solid tumors, *e.g.*, the human breast sample, possess the pathological characteristics of spatial continuity (Figure S3).^32^ Accordingly, both cancerous ROIs and para-carcinoma ROIs in the MSI data of the tumor sample are spatially contiguous. Especially, margins of sub-regions are supposed to be natural edges of sub-populations of tumor samples. In view of this situation, we construct a general mode spat-contig of dc-DeepMSI to separate ROIs of carcinoma from those of para-carcinoma. As expected, the MSI data is successfully segmented into two separate sub-regions with a clear boundary, namely, cancerous (blue) and para-carcinoma (light gray) regions (Figure 3), which shows good consistency with the results from morphological evaluation (Figure S3). The scatter plot shows that data points from the cancerous region (colored points) and data points from the para-carcinoma region (gray points) can be clearly separated from each other in the cubic embedding space (Figure 3m) or say the feature space of dimension reduced MSI data, which implies that molecular features are significantly different from each other between cancerous and para-carcinoma sub-regions and demonstrates the accuracy and efficiency of dc-DeepMSI in cancerous sub-region detection.

#### Tumor Intra-heterogeneity Exploration *via* the Specific spat-spor Mode

The human tumor has significant intra-heterogeneity in molecular phenotypes, microenvironment, and metabolic regulation.^33^ The morphological analysis of the invasive ductal carcinoma with neuroendocrine differentiation (NED) indicates that the breast tumor displays significant intra-tumor heterogeneity that is featured in the sporadic distribution between cancerous regions with different degrees in differentiation and stromal regions. As shown in Figure S3, at least two typical cancerous regions can be classified by using immunohistochemistry (IHC) analysis according to the chromogranin A expression, including the cancerous region with NED (18%) and cancerous region (15%) as well as respective typical invasive regions.

To explore the molecular phenotypes and microenvironments in the tumor sample, we build a model of dc-DeepMSI with the specific mode spat-spor on the MSI data of the human breast tumor sample. dc-DeepMSI cluster data points in the intact tumor sample into 10 different sub-regions (Figure 3b), in which most of the sub-regions are in agreement with the results of the morphological information. For example, three sub-regions are assigned and associated to two major molecular phenotypes (Figure 3c–e). Additionally, there is one invasive sub-region (Figure 3f) and six stromal sub-regions (Figure 3g-l). Herein, invasive ductal carcinoma with NED-related segmentation from an intact tumor sample is given in Figure 3c,d, showing the discrete imaging patterns with obscure boundary between the nests of neoplastic cells, which is basically consistent with morphological results. We also achieve the accurate invasive ductal carcinoma-related segmentation (Figure 3e). The results exhibit the clear boundary between the nests of neoplastic cells according to the morphological results. Continuously, a typical invasive region (Figure 3f) and stromal region (Figure 3j) are segmented from the intact sample, demonstrating the sporadic infiltration of neoplastic cells in the fibrous stroma as well as the randomness of spatial distribution of the stromal region, respectively.

The scatter plot shows that data points from the same sub-region gather together, while data points from different sub-regions are clearly separated from each other in the embedding space, which illustrates the distinct metabolic difference among the 10 sub-regions (Figure 3m). Scatter plots of the 10 sub-regions are shown in Figure S4. Violin plots display the distribution of Euclidean distances between data points of each sub-region and data points of para-carcinoma in the embedding space (Figure 3n). As we can expect, the data points of two molecular phenotype-related regions are far away from each other in the cubic embedding space, while data points of the stromal sub-regions are located in between the molecular phenotypes and the para-carcinoma, which indicates that lipid profiles of stromal sub-regions are more similar to para-carcinoma than the invasion and the two major molecular phenotype-related regions. The results show the ability of dc-DeepMSI in exploring metabolic heterogeneities from MSI data of the tumor sample.

### Screening of the Underlying Molecular Markers

The underlying molecular markers from ROIs can help us interpret and validate dc-DeepMSI segmentation results. As a traditional application scenario, MSI is capable of providing the spatial distribution of the marker by an expression of a single ion. Nevertheless, both multiple molecules and their interaction play an important role in complex biological regulations, making it difficult to use the expression of a single ion to elaborate the spatial heterogeneity of bio-samples. To solve this problem, a two-stage screening approach is used here to identify the molecular markers between two given ROIs, namely, the target ROI and control ROI. The screening approach is detailed in Material S2.

**Figure 4:**
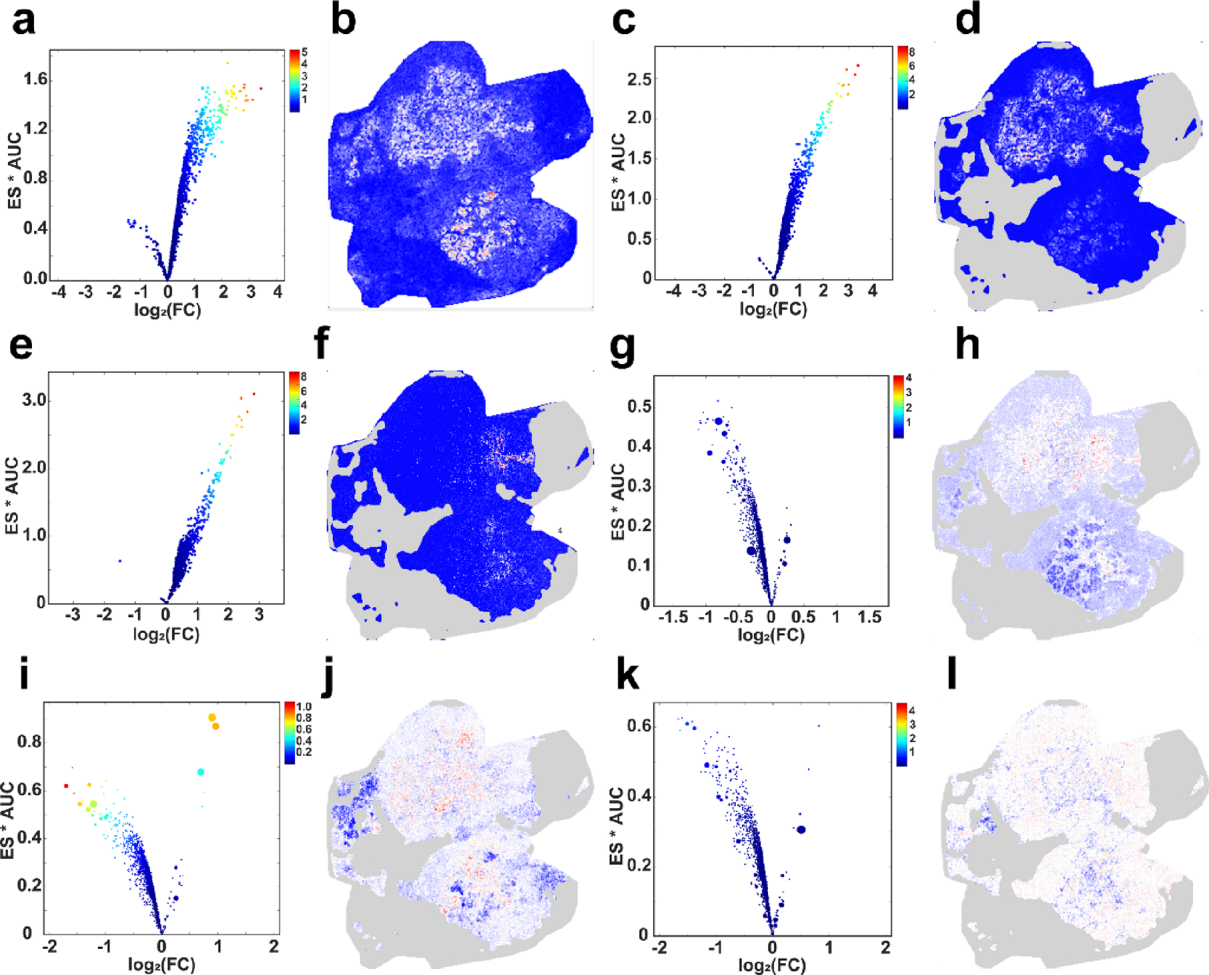
Volcano plots show three measures including ES, AUC, and log_2_(FC) between the target sub-region and the control region for all ions. Color-encoded ion’s images show the normalized abundances of selected markers or co-expressive ions. In volcano plots, the color represents the value of ES × AUC × | log_2_(FC)|, and the warmer the color, the larger the value. The point size in volcano plots represents the absolute LASSO regression coefficient, the larger the size, the bigger the absolute coefficient. The target and control regions in (a, b) are cancerous and para-carcinoma regions, respectively. The target sub-regions are (c, d) IDC-NED-1, (e, f) IDC-NED-2, (g, h) IDC, (i, j) invasive, (k, l) stromal, respectively, and the control region is the remaining sub-regions excluding the target sub-region.

The existing evidences have suggested that abundances and spatial distribution of lipids are expressed abnormally in human breast tumor tissues, with a close relationship to aggressiveness and metastatic potentials of tumors. Tumor cells can generate excess lipids to maintain metabolic supplies and support tumor proliferation and invasion.^34,35^ Taking the human breast tumor for instance, the two-stage screening approach is carried out on each sub-region (target ROI) with respect to the other nine sub-regions (control ROI) to identify the lipid markers or co-expressive lipid ions of the target ROI. Volcano plots and ion images are used to visualize the screening results (Figure 4). The lists including single- and multi-co-expressive lipid markers from the ROIs of the breast tumor sample can be found in Table S4.

According to the results of the two-stage screening approach, four lipid ions are found to be the lipid markers of carcinoma with respect to para-carcinoma regions. For example, the abundant *m/z* 743.65.73 PE (36:2), which is observed in cancerous regions corresponding to the H&E stain image, is absent in the para-carcinoma regions (Figure 4b). We find that eight lipids are upregulated in the specific sub-regions containing invasive ductal carcinoma with NED, such as *m/z* 839.98 PC (40:3) (Figure 4d) and *m/z* 795.89 PE (40:4) (Figure 4f).

In the tumor sample at the invasive ductal carcinoma, invasive, and stromal sub-regions, the results have shown that a series of ions jointly contribute to shape their own molecular profiles. For example, co-expression of 17 ions is accumulated in invasive ductal carcinoma-associated sub-regions (Figure 4h), which is equivalent to a complex marker with ES = 1.57, AUC = 0.87, and log_2_(FC) = 3.20. Similarly, co-expression of 10 ions is accumulated in invasive sub-regions (Figure 4j), which is equivalent to a complex marker with ES = 3.49, AUC = 0.94, and log_2_(FC) = 3.14. In addition, one of the stromal sub-regions is delineated by the co-expression of 30 ions (Figure 4l), which is equivalent to a complex marker with ES = 1.66, AUC = 0.83, and log_2_(FC) = 2.25. A more detailed result is available in Figure S5.

We have demonstrated the significant heterogeneity of spatial distribution of lipid markers in the form of single-ion expression and multi-ion co-expression by the proposed two-stage screening approach, which has an important conductive function to metabolic reprogramming of tumor progression.

## Conclusions

The screening and identification of metabolism-related sub-regions play an important role in better describing the molecular characteristics throughout the biological process and in optimizing the diagnosis and treatment of diseases.^36^ A previous study reported the potential of MSI for the discovery of metabolic heterogeneity in tumor tissues.^37^ Actually, the MSI dataset is appropriate for the metabolic heterogeneous analysis because MSI provides us with (1) very rich biological information from the molecular level, usually achieving thousands of compounds simultaneously, and (2) spatially resolved and (3) (relative) quantitative molecular information for *in situ* analysis of bio-samples. In this paper, for the first time, we introduce a divide-and-conquer strategy into a deep neural network and present a flexible dc-DeepMSI model to screen ROIs that are spatially sporadic or spatially contiguous from MSI datasets of complex bio-samples, like human tumor or mouse fetus. dc-DeepMSI provides the possibility to characterize the molecular phenotypes and biomarkers in human tumors and identifies sub-organs in mouse fetus based on spectral similarity and spatial closeness of targeted subpopulations.

The proposed model of dc-DeepMSI outperforms state-of-the-art MSI segmentation methods, which benefits from the following aspects: (1) The adoption of a divide-and-conquer strategy greatly reduces complexity of a deep learning model and improve the model stability. (2) The autoencoder-based dimensionality reduction leads to a stable and low-dimensional representation of MSI data while minimizing information loss. (3) Feature clustering using two structurally identical but randomly initialized CNNs achieves a robust segmentation, in which the two CNNs work in an adversarial and collaborative way. Moreover, two temporally ensemble CNNs stabilize effectively the segmentation. (4) To deal with two representatives ROI scenarios, namely, spatially contiguous ROIs or spatially sporadic ROIs, dc-DeepMSI designs the general mode of spat-contig and the specific mode of spat-spor to meet with different ROI scenarios in a variety of specimens. (5) Since dc-DeepMSI utilizes no prior knowledge or characteristics of lipid molecules, it is also applicable to other types of molecules. Several results have proven that dc-DeepMSI is a straightforward and more robust approach to identify the presence of sub-regions characterized by similar mass spectrometry profiles, providing results that are not captured by histological technologies.

We provide in this paper a deep learning-based method to identify underlying metabolic heterogeneity from high-dimensional MSI data. The proposed model is also expected to be broadly applicable in multiple computational tasks with hyperspectral imaging techniques, such as microscopy imaging, remote sensing imaging, and other medical imaging. We believe that our work will facilitate the extensive applications of unsupervised deep learning on high-dimensional data analysis.

## Data Availability

Python code of dc-DeepMSI is available at GitHub: https://github.com/BioNet-XMU/dc-DeepMSI.

## Supporting Information Available

Clustering results obtained from different methods; architecture of two other deep learning models; H&E image of the human breast sample with 0.4× amplification; scatter plots of data points in embedding space corresponding for different sub-regions; molecular markers among sub-regions identified by dc-DeepMSI; detailed information of two MSI datasets; computational times of four different segmentation methods; parameters number for Kim *et al*.’s model and an end-to-end architecture model; list of lipid markers screened from the breast tumor sample; and details of experiments, lipid ion screening method, architecture of the dc-DeepMSI model, training strategy, model implementation, anti-noise ability evaluation, and model stability evaluation (PDF)
