# Supplementary material for: Divide and Conquer: A Flexible Deep Learning Strategy for Exploring Metabolic Heterogeneity from Mass Spectrometry Imaging Data
Source: Anal Chem. 2023 Jan 12;95(3):1924–32. doi: 10.1021/acs.analchem.2c04045 (PMC9878502; doi:10.1021/acs.analchem.2c04045)
Supplement: Supplementary file 1 — ac2c04045_si_001.pdf [file ac2c04045_si_001.pdf]

Supporting Information

Divide-and-conquer: a flexible deep learning strategy for exploring metabolic heterogeneity from mass spectrometry imaging data

Lei Guo [a,#], Jiyang Dong [a,#,*], Xiangnan Xu [d], Zhichao Wu [e], Yinbin Zhang [f], Yongwei Wang [g], Pengfei Li [g], Zhi Tang [h], Chao Zhao [b,c,*], Zongwei Cai [c,*]

[a] National Institute for Data Science in Health and Medicine, Department of Electronic Science, Xiamen University, Xiamen 361005, China

[b] Bionic Sensing and Intelligence Center, Institute of Biomedical and Health Engineering, Shenzhen Institute of Advanced Technology, Chinese Academy of Sciences, Shenzhen 518055, China

[c] State Key Laboratory of Environmental and Biological Analysis, Department of Chemistry, Hong Kong Baptist University, Hong Kong SAR 999077, China

[d] School of Mathematics and Statistics, The University of Sydney, NSW 2006, Australia.

[e] School of Artificial Intelligence, Beijing Normal University, Beijing 100875, China.

[f] Department of Oncology, The Second Affiliated Hospital of Medical College, Xi'an Jiaotong University, Shaanxi 710004, China

[g] Bruker Scientific Technology Co., Ltd., Beijing 100086, China

[h] School of Public Health, Dongguan Key Laboratory of Environmental Medicine, Institute of Environmental Health, Guangdong Medical University, Dongguan 523808, China

This supplementary file includes:

1. Supplementary Figures

Figure S1. Clustering results obtained from different methods. S3
Figure S2. Architectures of two deep learning models. S4
Figure S3. The H&E image of human breast sample with 0.4 × amplification...........S5
Figure S4. Scatter plots of data points in embedding space corresponding for different sub-regions. S6
Figure S5. Molecular markers among sub-regions identified by dc-DeepMSI. S7

2. Supplementary Tables

Table S1. Detailed information of two MSI datasets...S8
Table S2. Computational times of four different segmentation methods. S8
Table S3. Parameters number for Kim's model and an end-to-end architecture model. S9
Table S4. The list of lipid markers screened from breast tumor sample. S10

3. Supplementary Materials

Material S1. Detailed of experiments S11
Material S2. Detailed of Lipid ions screening method S12
Material S3. The architecture of dc-DeepMSI..S15
Material S4. Training strategy and implementation. S17
Material S5. Anti-noise ability evaluation S19
Material S6. Model stability evaluation S21

References S23

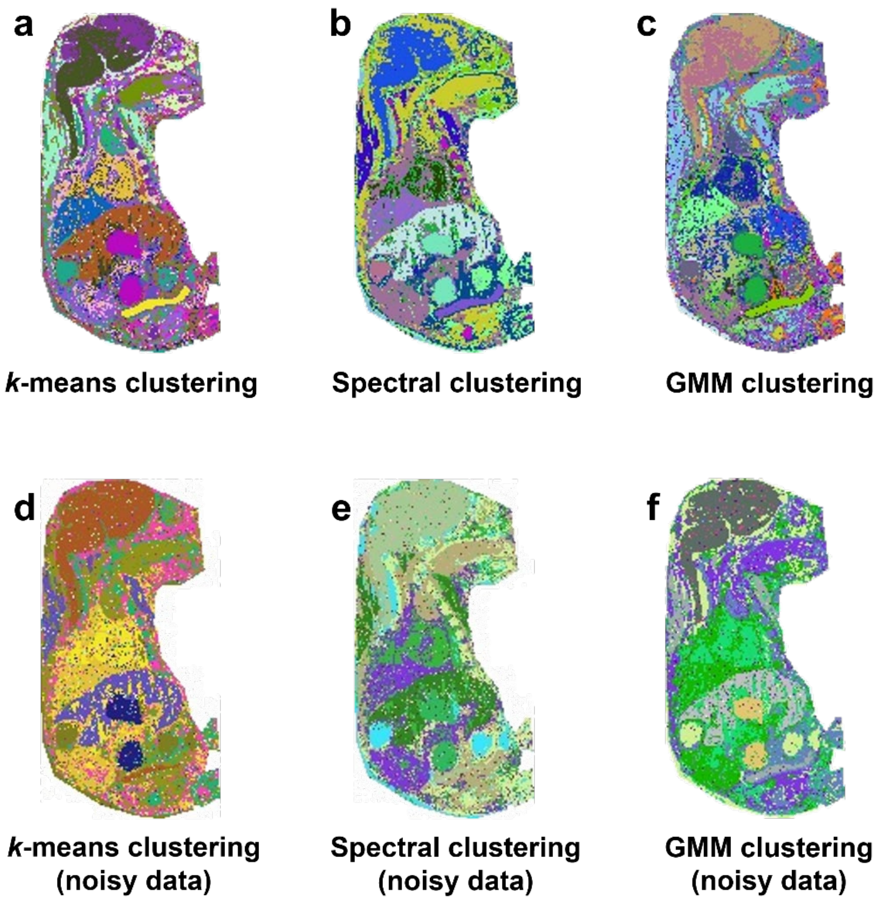

Figure S1. Clustering results obtained from different methods. (a-c) Original data: (a) *k*-means clustering, (b) Spectral clustering, (c) Gaussian mixture model (GMM) clustering. (d-f) Noisy data: (d) *k*-means clustering, (e) Spectral clustering, (f) GMM clustering.

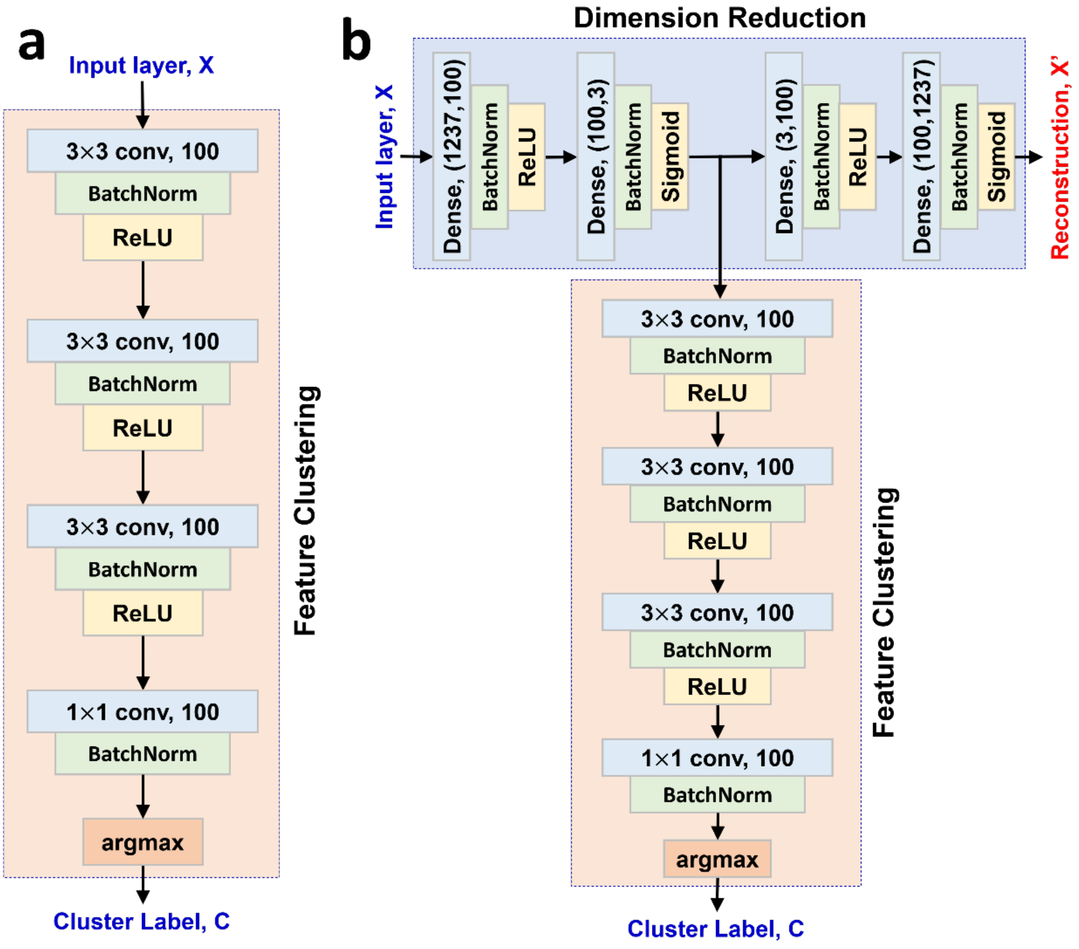

Figure S2. Architectures of two deep learning models. (a) An end-to-end structure model; (b) Kim's model. Note: Parameters number for each layer can refer to Table S1.

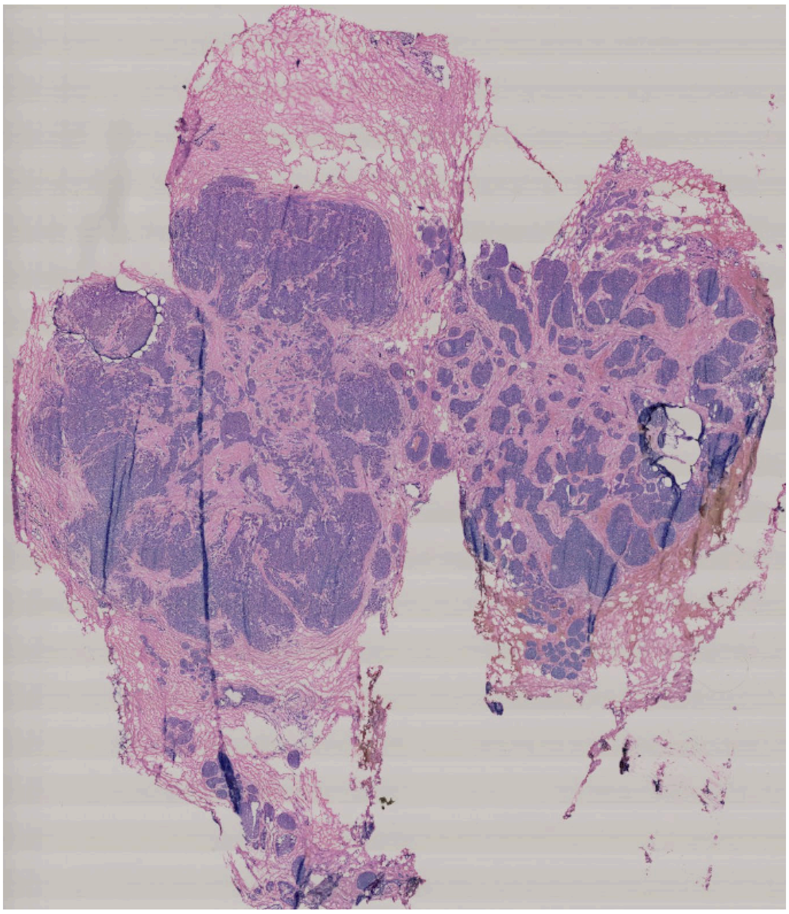

Figure S3. The H&E image of human breast sample with 0.4 × amplification.[1]

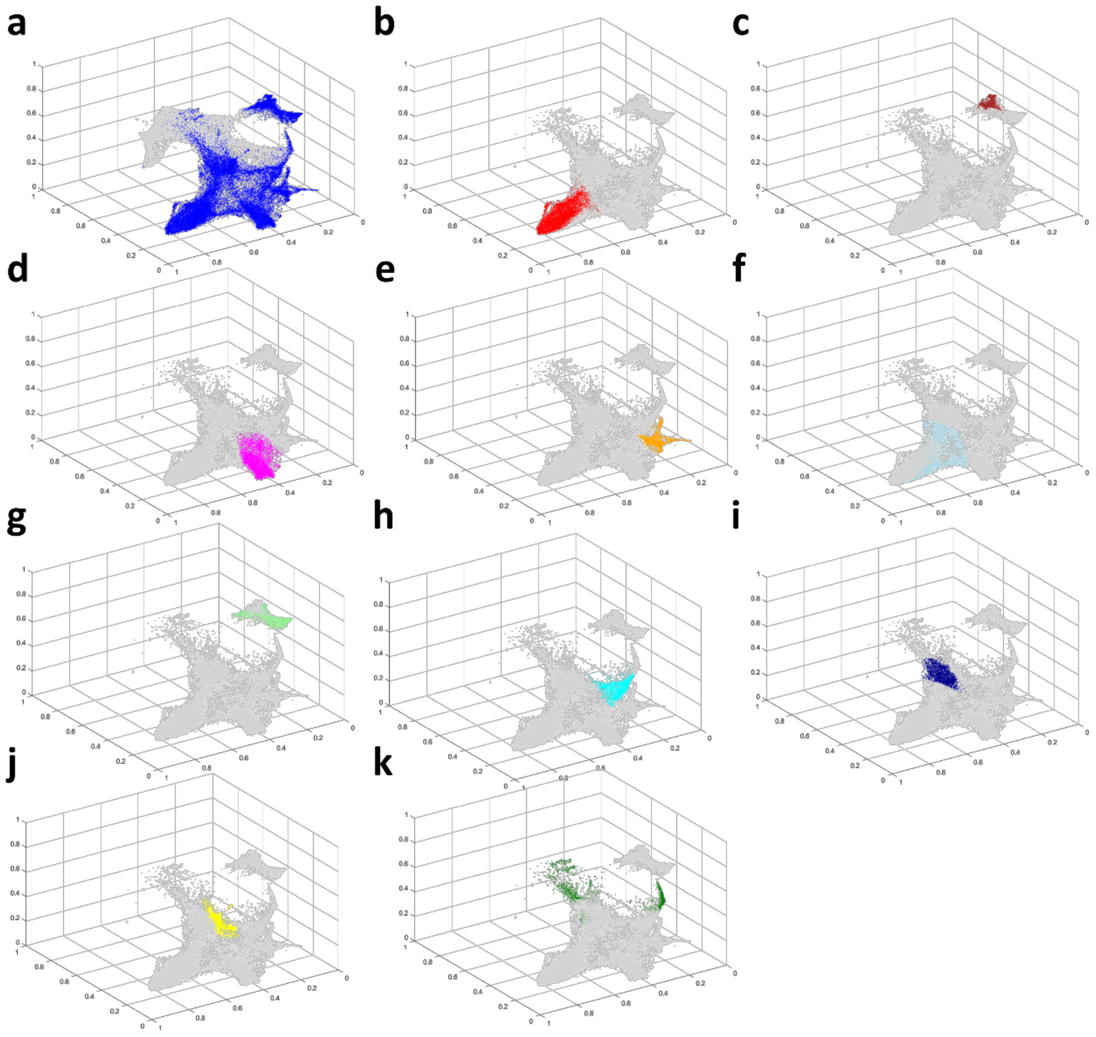

Figure S4. Scatter plots of data points in embedding space corresponding for different sub-regions. (a) Cancerous (blue) and para-carcinoma (light gray) regions. (b) IDC-NED-1 (red). (c) IDC-NED-2 (brown). (d) IDC (magenta). (e) Invasion (orange). (f) Stromal 1 (light blue). (g) Stromal 2 (light green). (h) Stromal 3 (cyan). (i) Stromal 4 (dark blue). (j) Stromal 5 (yellow). (k) Stromal 6 (green).

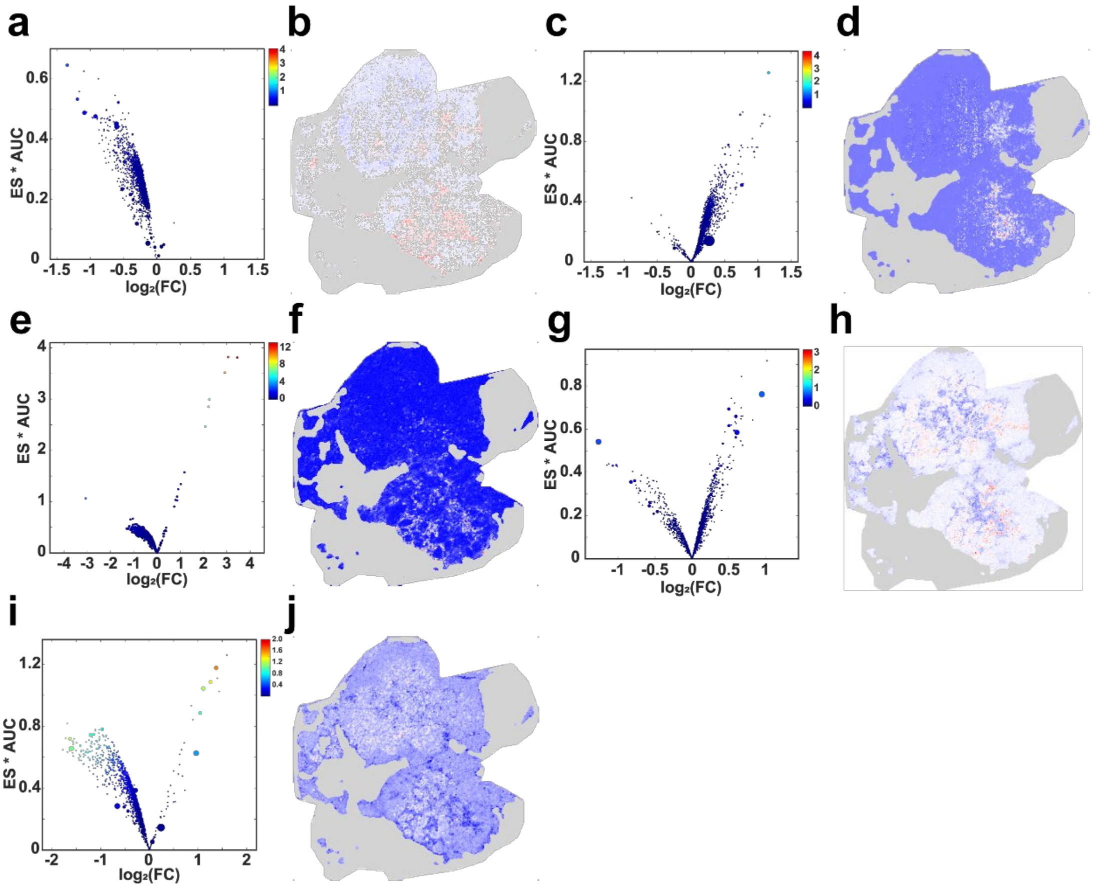

Figure S5. Molecular markers among sub-regions identified by dc-DeepMSI. The volcano plots show three measures including ES, AUC and $\log_2(\mathrm{FC})$ between the target sub-region and the control region for all ions. Color encoded ion's images show the normalized abundances of selected markers or co-expressive ions. In volcano plots, the color represents the value of $\mathrm{ES} * \mathrm{AUC} * |\log_2(\mathrm{FC})|$, and the warmer the color, the larger the value. The point size in volcano plots represents the absolute LASSO regression coefficient, the larger the size, the bigger the absolute coefficient. The target sub-region is (a, b) stromal 1; (c, d) stromal 2; (e, f), stromal 3; (g, h) stromal 4; (i, j) stromal 6; respectively, and the control region is the remain sub-region excluded the target sub-region.

Table S1. Detailed information of two MSI datasets

MSI data	Size	Spectra number	Data points	mass range
Mouse fetus	2.9GB	16903	4300	200-1600 *m/z*
Human breast cancer	5.51GB	67521	21900	400-1100 *m/z*

Table S2. Computational times of four different segmentation methods.

**Method**	**Running Time**
t-SNE + *k*-means	44 min
SCiLS Lab	34 min
Cardinal	45 min
dc-DeepMSI	27 min

Table S3. Parameters number for Kim's model and an end-to-end architecture model.

Method	Module	Layer Name	Output Size	Parameter Number
Kim's model (446120)	Dimension Reduction	Input layer	(21614, 1237)	0
		FC_1 (ReLU)	(21614, 100)	123,800
		BatchNorm	(21614, 100)	200
		FC_2 (Sigmoid)	(21614, 3)	303
		BatchNorm	(21614, 3)	6
		FC_3 (ReLU)	(21614, 100)	400
		BatchNorm	(21614, 100)	200
		FC_4 (Sigmoid)	(21614, 1237)	124,937
		BatchNorm	(21614, 1237)	2,474
	Feature Clustering	Conv_1 (3×3, 100, ReLU)	(1, 100, 202, 107)	2,800
		BatchNorm	(1, 100, 202, 107)	200
		Conv_2 (3×3, 100, ReLU)	(1, 100, 202, 107)	90,100
		BatchNorm	(1, 100, 202, 107)	200
		Conv_3 (3×3, 100, ReLU)	(1, 100, 202, 107)	90,100
		BatchNorm	(1, 100, 202, 107)	200
		Conv_4 (1×1, 100, ReLU)	(1, 100, 202, 107)	10,000
		BatchNorm	(1, 100, 202, 107)	200
End-to-end architecture model (1304400)	Feature Clustering	Input layer	(1, 1237, 202, 107)	0
		Conv_1 (3×3, 100, ReLU)	(1, 100, 202, 107)	1,113,400
		BatchNorm	(1, 100, 202, 107)	200
		Conv_2 (3×3, 100, ReLU)	(1, 100, 202, 107)	90,100
		BatchNorm	(1, 100, 202, 107)	200
		Conv_3 (3×3, 100, ReLU)	(1, 100, 202, 107)	90,100
		BatchNorm	(1, 100, 202, 107)	200
		Conv_4 (1×1, 100, ReLU)	(1, 100, 202, 107)	10,000
		BatchNorm	(1, 100, 202, 107)	200

Table S4. The list of lipid markers screened from breast tumor sample.

[M-H]-	log_2(FC)	AUC	Hedges ES	LASSO Weight
615.1503	0.817	0.649	0.9298	0.0085
616.1667	0.483	0.610	0.5752	0.0543
616.4530	-0.488	0.632	0.4967	0.0060
634.3908	-0.581	0.646	0.5317	0.1066
687.5877	-0.909	0.660	0.5920	0.1306
688.5890	-0.812	0.666	0.5975	0.0818
726.4544	0.067	0.533	0.1125	-0.0270
734.6307	-0.741	0.693	0.6468	0.0115
740.6469	-0.978	0.689	0.6479	0.0125
751.7047	-0.011	0.509	0.0146	-0.0610
768.7212	-1.160	0.716	0.6856	-0.3300
769.7286	-0.998	0.710	0.6850	0.1244
773.7169	-0.639	0.638	0.5216	0.0348
788.7174	-3.951	0.980	1.1983	0.1943
788.7527	-0.136	0.508	0.1149	-0.1955
797.8116	-0.630	0.669	0.5950	-0.0132
799.7509	-0.467	0.640	0.5096	0.0256
812.7970	-0.606	0.606	0.4499	0.2420
822.8680	-0.751	0.704	0.6731	0.1061
846.9476	-0.602	0.674	0.6072	0.0258
883.9288	-0.301	0.576	0.3057	0.1651
885.9943	0.504	0.630	0.4846	-1.0000
889.0114	-0.798	0.651	0.5445	0.0032
890.0327	-1.509	0.788	0.7730	-0.178
890.1829	-0.025	0.522	0.0557	-0.1688
890.8976	0.046	0.528	0.0887	-0.3315
891.0512	-1.384	0.777	0.7681	-0.1989
892.9586	-0.046	0.517	0.0854	-0.1722
916.0227	0.007	0.508	0.0130	-0.0786
917.0221	-0.021	0.506	0.0387	-0.0031

Material S1. Detailed of experiments

All of mice are treated humanely with the consideration of alleviating suffering. Six-week-old C57BL/6 male and female mice are housed under specific pathogen free condition with controlled temperature, humidity and 12 hrs dark: light cycle. One male and two females are bred and observed by a vaginal plug. And then, females are placed in a separate cage after successful mating. The whole-body mouse fetuses at embryonic day 18 are collected for MALDI-MSI analysis.

Human samples were collected from tumor cores and tumor margins during surgical oncology procedures. The tumor sample was an invasive ductal carcinoma (48 years old) with neuroendocrine differentiation.

Material S2. Detailed of Lipid ions screening method

Hedges' g is a measure of effect size (ES) that tells us how much one ROI differs from another, which can be calculated as,

$$\text{Hedges}'\ \text{g} = \frac{|\mu_1 - \mu_2|}{\sigma^*_{pooled}} \tag{1}$$

where μ_1, μ_2 are the mean abundances of the target ROI and the control ROI respectively, and σ^*_{pooled} is the pooled and weighted standard deviation,

$$\sigma^*_{pooled} = \sqrt{\frac{(n_1 - 1)\sigma_1^2 + (n_2 - 1)\sigma_2^2}{n_1 + n_2 - 1}} \tag{2}$$

where σ_1 and σ_2 are the standard deviations of the target ROI and the control ROI respectively. The larger the effect size, the greater the difference between two ROIs.

Fold-change (FC) is used to evaluate the abundance difference between two given ROIs, which is calculated as follows:

$$\text{FC} = \frac{\mu_1}{\mu_2} \tag{3}$$

where μ_1, μ_2 are the mean abundances of the target ROI and the control ROI respectively.

The area under the curve (AUC) is a measure of the ability of a classifier to distinguish between classes and is used as a summary of the receiver operating characteristic (ROC) curve. The higher the AUC, the better the performance of the model at distinguishing between the positive and negative classes. Here the positive and negative classes are the target and control ROIs respectively, and logistic regression is adopted to be the classifier model.

Furthermore, least absolute shrinkage and selection operator (LASSO) regression is used to identify co-expressive lipid ions for the target ROI with respect to the

control ROI.

Let $X_{N\times P}$ be the data matrix of two given ROIs with N data points (pixels) and P lipid ions in each pixel, y be the ROI belonging vector of the N pixels. We can build a linear regression model on (X, y) as follows,

$$y = \beta_0 + \beta X + \mathcal{E} \tag{4}$$

where $\beta = (\beta_1, \beta_2, \cdots, \beta_P)$ is the regression coefficients, and $\mathcal{E}$ is the residuals errors. Impose LASSO penalty on the optimization of β, we have

$$\hat{\beta}^{lasso} = \underset{\beta}{\operatorname{argmin}} \left\{ \frac{1}{2} \sum_{i=1}^{N} (y_i - \beta_0 - \sum_{j=1}^{P} x_{ij}\beta_j)^2 + \lambda \sum_{j=1}^{P} |\beta_j| \right\} \tag{5}$$

Then most of the regression coefficients will be zero.

The lipid ions of non-zero regression coefficients are defined as the co-expressive lipid ions of the target ROI, which acts as a lipid marker.

A two-stage screening approach is used here to identify the molecular markers between two given ROIs, namely target ROI and control ROI. The first stage uses three univariate statistics to quantify the difference of abundance of a ion between target and control ROIs, that is, fold-change (FC), area under the receiver operating characteristic curve (AUC) and Hedges'g effect size (ES). Then ions are defined as markers of the target ROI with respect to control ROI if they satisfy with the criteria as follows,

$$(\mathrm{ES} \times \mathrm{AUC}) \geq 1.5 \text{ and } |\log_2 \mathrm{FC}| \geq 1. \tag{6}$$

If no marker is found in the first stage, we continue the second stage.

The second stage builds a linear regression model on the abundance matrix X and the ROIs belonging vector y vector,

$$y = \beta_0 + \beta X + \mathcal{E} \tag{7}$$

where $\beta = (\beta_1, \beta_2, \cdots, \beta_i, \cdots)$ is the regression coefficients for ions, and $\mathcal{E}$ is the residuals errors. By imposing a least absolute shrinkage and selection operator (LASSO) penalty on the optimization of β, only a few ions are of non-zero coefficients, then these ions are defined as the co-expressive ions of the target ROI, which acts as a marker.

Material S3. The architecture of dc-DeepMSI

dc-DeepMSI is consisted of two modules, *i.e.*, dimensionality reduction (DR-module) and feature clustering (FC-module), as shown in **Figure 1a**. DR-module is to learn a nonlinear mapping $f(\cdot|\vartheta)$ to project the high-dimensional data $\mathrm{X}_{M\times\mathrm{N}\times H}$ into a low-dimensional data $\mathrm{Y}_{M\times N\times L}$ as follows,

$$\mathrm{Y}_{M\times N\times L} = f(\mathrm{X}_{M\times\mathrm{N}\times H}|\vartheta) \tag{8}$$

where ϑ is the network parameters in DR-module to be trained. FC-module is to learn a nonlinear mapping function $g(\cdot|\theta)$ from $\mathrm{Y}_{M\times N\times L}$ to segmentation map/cluster label $\mathrm{C}_{M\times N}$ as follows,

$$\mathrm{C}_{M\times N} = g(\mathrm{Y}_{M\times N\times L}|\theta) \tag{9}$$

where θ is the network parameters in FC-module to be trained.

To achieve the nonlinear mapping, FC-module is designed with two parallel feature extraction (FE) blocks and two temporally ensemble FE blocks, as shown in **Figure 1a**.

Firstly, each FE block is implemented by a CNN of n components and a linear classifier (**Figure 1b**), in which the CNN component is consisted of a 2D convolutional layer of p channels and $s\times s$ kernel size, a batch normalization layer and a ReLU activation function, while the linear classifier is consisted of a 2D convolutional layer of q filters and 1×1 kernel size. The output of FE block is a response map $\mathrm{R}_{M\times N\times q} = \left(r_{m,n,i}\right)$, on which a segmentation map, or say cluster label $\mathrm{C}_{M\times N} = \left(C_{m,n}\right)$, will be produced by applying *argmax* classifying,

$$C_{m,n} := \left\{i \middle| r_{m,n,i} \geq r_{m,n,j}, \forall j \neq i \leq q\right\} \tag{10}$$

Secondly, the temporally ensemble FE block is accomplished by averaging the parameters of its corresponding FE block at each iteration t as follows,

$$\theta^E(t) = \alpha \cdot \theta^E(t-1) + (1-\alpha) \cdot \theta(t) \tag{11}$$

where $\theta(t)$ and $\theta^E(t)$ are the parameters of FE block and its corresponding temporally ensemble FE block at time t, and $0 \leq \alpha < 1$ is the ensemble momentum.

Specifically, the two FE blocks and two ensemble FE blocks map the input $Y_{M\times N\times L}$ to 4-different segmentation maps as,

$$\begin{cases} C^1_{M\times N} = g^1(Y_{M\times N\times L}|\theta^1) \\ C^2_{M\times N} = g^2(Y_{M\times N\times L}|\theta^2) \\ C^{1E}_{M\times N} = g^{1E}(Y_{M\times N\times L}|\theta^{1E}) \\ C^{2E}_{M\times N} = g^{2E}(Y_{M\times N\times L}|\theta^{2E}) \end{cases} \tag{12}$$

The four FE blocks work adversarially and collaboratively to achieve a final segmentation map $C_{M\times N}$.

Material S4. Training strategy and implementation.

Divide-and-conquer strategy is designed to train the DR module and FC module, respectively. DR-module is implemented by an autoencoder framework, which is consisted of two blocks, i.e., the encoder block and the decoder block as follows,

$$\mathrm{Y}_{M\times N\times L} = f(\mathrm{X}_{M\times N\times H}|\vartheta) \tag{13}$$

$$\mathrm{X'}_{M\times N\times H} = f^{d}(\mathrm{Y}_{M\times N\times L}|\vartheta^{d}) \tag{14}$$

where f and f^d are the mapping functions of encoder and decoder, ϑ and ϑ^d are the parameter of encoder and decoder blocks respectively. $\mathrm{Y}_{M\times N\times L}$ is the reduced data. We use a loss function $\mathcal{L}_{rec}$ to train the autoencoder module as follows,

$$\mathcal{L}_{rec} = \frac{1}{M\times N}\sum_{m=1}^{M}\sum_{n=1}^{N} 1 - \left(\frac{\mathrm{X}_{m,n}\cdot \mathrm{X'}_{m,n}}{\|\mathrm{X}_{M\times N}\|_2 \cdot \|\mathrm{X'}_{M\times N}\|_2}\right) \tag{15}$$

where $\|\cdot\|_2$ is l_2-norm.

The loss function $\mathcal{L}$ in FC-module is a weighted combination of three parts as,

$$\begin{aligned}\mathcal{L} &= \omega_1 \cdot (\mathcal{L}_{sim}(\mathrm{R}^1,\mathrm{C}^1) + \mathcal{L}_{sim}(\mathrm{R}^2,\mathrm{C}^2)) \\ &+ \omega_2 \cdot \left(\mathcal{L}_{sta}(\mathrm{R}^1,\mathrm{C}^{2E}) + \mathcal{L}_{sta}(\mathrm{R}^2,\mathrm{C}^{1E})\right) \\ &+ \omega_3 \cdot (\mathcal{L}_{TV}(\mathrm{R}^1)+\mathcal{L}_{TV}(\mathrm{R}^2))\end{aligned} \tag{16}$$

where $\omega_1, \omega_2, \omega_3$ are combinational weights, $\mathcal{L}_{sim}$, $\mathcal{L}_{sta}$, and $\mathcal{L}_{TV}$ are three loss functions to optimize the network parameters.

Firstly, the similarity loss of $\mathcal{L}_{sim}$ is to make pixels with similar features be assigned to same cluster, which is designed based on cross entropy between the response map R and segmentation map C as follows,

$$\mathcal{L}_{sim}(\mathrm{R},\mathrm{C}) = \frac{1}{M\times N}\sum_{m=1}^{M}\sum_{n=1}^{N}\sum_{i=1}^{q} -\delta\left(i - C_{m,n}\right)\cdot \ln\, r_{m,n,i} \tag{17}$$

where

$$\delta(t) = \begin{cases} 1, & \text{if } t = 0 \\ 0, & \text{Otherwise} \end{cases}$$

Secondly, the stability loss of $\mathcal{L}_{sta}$ is to stabilize the segmentation result, which is calculated using to the response map of one FE model (R) and the segmentation map of the temporally average of another FE block $(\mathrm{C}^{\sim E})$ as follows:

$$\mathcal{L}_{sta}(R, \mathrm{C}^{\sim E}) = \frac{1}{M \times N} \sum_{m=1}^{M} \sum_{n=1}^{N} \max\left(0, r_{m,n}^{\mathrm{neg}} - r_{m,n}^{\mathrm{pos}} + \alpha\right) \tag{18}$$

where α is a margin parameter, and

$$r_{m,n}^{\mathrm{neg}} := \left\{r_{i,j} \left| min\left(\left\|r_{m,n} - r_{i,j}\right\|_2^2\right), \forall\, C_{i,j}^{\sim E} \neq C_{m,n}^{\sim E} \;\&\; m,n \neq i,j\right.\right\} \tag{19}$$

$$r_{m,n}^{\mathrm{pos}} := \left\{r_{i,j} \left| max\left(\left\|r_{m,n} - r_{i,j}\right\|_2^2\right), \forall\, C_{i,j}^{\sim E} = C_{m,n}^{\sim E} \;\&\; m,n \neq i,j\right.\right\} \tag{20}$$

Thirdly, the total variation (TV) loss of $\mathcal{L}_{TV}$ is to make pixels of spatially close be in a same cluster, which is used to decrease the differences between neighboring pixels,

$$\mathcal{L}_{TV}(\mathrm{R}) = \frac{1}{M \times N} \sum_{m=1}^{M-1} \sum_{n=1}^{N-1} \left\|r_{m+1,n} - r_{m,n}\right\|_1 + \left\|r_{m,n+1} - r_{m,n}\right\|_1 \tag{21}$$

where $\|\cdot\|_1$ is l_1-norm.

Stochastic gradient descent optimizer is adopted to train both DR-module and FC-module, where the learning rate and the momentum are set to be 0.01 and 0.9 respectively. Network parameters are initialized to be normal distribution $N(0,0.02)$. The proposed model is implemented in Python with PyTorch library and trained the models on a workstation equipped with a GPU Nvidia GTX 2080Ti graphics card.

Material S5. Anti-noise ability evaluation

Previous studies have shown that noise in MSI data is likely to be governed by Poisson distribution.[2] To evaluate the robustness against noise of different deep models, Poisson distribution noise $\boldsymbol{\mathcal{N}} \sim \mathrm{P}(\lambda)$ is simulated and added on the original data as follows,

$$\mathbf{X}^{noisy} = \mathbf{X} + \boldsymbol{\mathcal{N}} \quad (22)$$

where $\mathbf{X}$ is the original data. The noise $\boldsymbol{\mathcal{N}}$ can be added on either the raw MSI data or the dimension reduced data depending on the experiment purpose. In this work, we set the parameter $\lambda = \Gamma(0.1 \cdot \mathbf{X})$, where $\Gamma(\cdot)$ is the Gamma function.

We show in main text that dc-DeepMSI is of better robustness than t-SNE+k-means against noise in the case of mouse fetus data segmentation (**Figure 2e, 2f**). For example, most of organs/sub-organs, especially for brain and its sub-organs, are still accurately segmented from the noisy data $\mathbf{X}^{noisy}$ by dc-DeepMSI (**Figure 2e**).

In addition, we find that the reason why dc-DeepMSI is of good robustness mainly owe to its double-CNN architecture in the feature clustering module. To illustrate the point, we present here some more results on noisy data segmentation. Three commonly used clustering methods including *k*-means, spectral clustering, gaussian mixture model (GMM) clustering are implemented to compare with the feature clustering module of dc-DeepMSI.

To be fair, we apply dc-DeepMSI on the high-dimensional MSI data of fetus mouse to obtain a low-dimensional data $\mathbf{X}$ by using the dimension reduction module only, then we generate and add Poisson noise on it to get the noisy data $\mathbf{X}^{noisy}$. Three clustering methods are used to segment the noisy data $\mathbf{X}^{noisy}$, and the results are shown in **Figure S1**. Differing from dc-DeepMSI, the three methods deliver noisy

segmentation maps on which lots of small and isolated clusters can be seen from the result figures. These results imply that the double-CNN structured feature clustering module mainly contribute to the anti-noise ability of dc-DeepMSI.

Material S6. Model stability evaluation

Adjusted rand index (ARI) is used to evaluate model stability. Let M and M' be two segmentation maps from a same model with different parameters initializations, then the ARI of the model can be defined by,[3]

$$\mathrm{ARI}(M', M) = \frac{\sum_{ij}\binom{N_{ij}}{2} - [\sum_i \binom{N_i}{2} \cdot \sum_j \binom{N_j}{2}]/\binom{N}{2}}{0.5 \cdot [\sum_i \binom{N_i}{2} + \sum_j \binom{N_j}{2}] - [\sum_i \binom{N_i}{2} \cdot \sum_j \binom{N_j}{2}]/\binom{N}{2}} \qquad (23)$$

Here, N is the number of data points in a given data set and N_{ij} is the number of data points of the class label $C_j' \in M'$ assigned to cluster C_i in segmentation map M. N_i is the number of data points in cluster C_i of segmentation map M, and N_j is the number of data points in class C_j'. In general, an ARI value lies between 0 and 1. The index value is equal to 1 only if a partition is completely identical to the intrinsic structure and close to 0 for a random partition.

Taking fetus mouse data as an example, we illustrate here that the main reasons why dc-DeepMSI is of good model ability lie in the divide-and-conquer strategy and the parallel-CNN based feature clustering module. Two deep learning models are constructed to compare with dc-DeepMSI on model stability. One is an end-to-end structure model which has no explicit dimension reduction module. Architecture of the end-to-end model is shown in **Figure S2a**. The other is a deep model constructed according to Kim's work.[4] Similar to dc-DeepMSI, both dimension reduction and feature clustering modules are explicitly designed in Kim's model, but differing from dc-DeepMSI, feature clustering module is implemented by a single-CNN structure in Kim's model (**Figure S2b**). Experimental results of 200 repetitions on parameter initializations show that Kim's model is of better ARI values than the end-to-end model, which imply that a deep model can benefit from the separate architecture design of dimension reduction module and feature clustering module to improve the

model stability (**Figure 2g**). In addition, the introduction of divide-and-conquer strategy could greatly reduce the number of parameters, which is another reason to enhance the model stability (**Table S1**). **Figure 2g** shows that dc-DeepMSI outperforms Kim's model with higher ARI values, which means that the parallel-CNN structure in feature clustering can further improve the model stability, which is consistent with the previous research.[5]

References

1 Zhao, C.; Yong, T.; Zhang, Y.; Xiao, Y.; Jin, Y.; Zheng, C.; Nirasawa, T.; Cai, Z. Breast cancer proliferation and deterioration-associated metabolic heterogeneity changes induced by exposure of bisphenol S, a widespread replacement of bisphenol A. J. Hazard. Mater. **2021**, 414, 125391.

2 Keenan, M. R.; Kotula, P. G. Accounting for Poisson noise in the multivariate analysis of ToF-SIMS spectrum images. *Surf. Interrface. Anal.,* **2004**, 36, 203-212.

3 Steinley, D. Properties of the Hubert-Arabie adjusted rand index. Psychological Methods., **2004**, 9, 386-396.

4 Kim, W.; Kanezaki, A.; Tanaka, M. Unsupervised Learning of Image Segmentation Based on Differentiable Feature Clustering. *IEEE. T. Image. Process.,* **2020**, 29, 8055-8068.

5 Yao, H.; Zhang, X.; Zhou, X.; Liu, S. Parallel Structure Deep Neural Network Using CNN and RNN with an Attention Mechanism for Breast Cancer Histology Image Classification. *Cancers.,* **2019**, 11,1901.
